# Laser-targeted ablation of the zebrafish embryonic ventricle: A novel model of cardiac injury and repair^☆^^☆☆^^★^

**DOI:** 10.1016/j.ijcard.2013.06.063

**Published:** 2013-10-09

**Authors:** Gianfranco Matrone, Jonathan M. Taylor, Kathryn S. Wilson, James Baily, Gordon D. Love, John M. Girkin, John J. Mullins, Carl S. Tucker, Martin A. Denvir

**Affiliations:** a University/BHF Centre for Cardiovascular Science, The Queen's Medical Research Institute, The University of Edinburgh, Edinburgh, EH16 4TJ, United Kingdom; b Centre for Advanced Instrumentation, Department of Physics, Durham University, South Road, Durham, DH1 3LE, United Kingdom; c Biophysical Science Institute, Department of Physics, Durham University, South Road, Durham, DH1 3LE, United Kingdom

**Keywords:** Zebrafish, Heart, Laser, Injury, Repair

## Abstract

**Background:**

While the adult zebrafish (*Danio rerio*) heart demonstrates a remarkable capacity for self-renewal following apical resection little is known about the response to injury in the embryonic heart.

**Methods:**

Injury to the beating zebrafish embryo heart was induced by laser using a transgenic zebrafish expressing cardiomyocyte specific green fluorescent protein. Changes in ejection fraction (EF), heart rate (HR), and caudal vein blood flow (CVBF) assessed by video capture techniques were assessed at 2, 24 and 48 h post-laser. Change in total and mitotic ventricular cardiomyocyte number following laser injury was also assessed by counting respectively DAPI (VCt) and Phospho-histone H3 (VCm) positive nuclei in isolated hearts using confocal microscopy.

**Results:**

Laser injury to the ventricle resulted in bradycardia and mild bleeding into the pericardium. At 2 h post-laser injury, there was a significant reduction in cardiac performance in lasered-hearts compared with controls (HR 117 ± 11 vs 167 ± 9 bpm, p ≤ 0.001; EF 14.1 ± 1.8 vs 20.1 ± 1.3%, p ≤ 0.001; CVBF 103 ± 15 vs 316 ± 13μms^− 1^, p ≤ 0.001, respectively). Isolated hearts showed a significant reduction in VCt at 2 h post-laser compared to controls (195 ± 15 vs 238 ± 15, p ≤ 0.05). Histology showed necrosis and apoptosis (TUNEL assay) at the site of laser injury. At 24 h post-laser cardiac performance and VCt had recovered fully to control levels. Pretreatment with the cell-cycle inhibitor, aphidicolin, significantly inhibited functional recovery of the ventricle accompanied by a significant inhibition of cardiomyocyte proliferation.

**Conclusions:**

Laser-targeted injury of the zebrafish embryonic heart is a novel and reproducible model of cardiac injury and repair suitable for pharmacological and molecular studies.

## 1 Introduction

The adult mammalian heart has little or no proliferative capacity [1,2] while the neonatal mouse heart retains the capacity to regenerate for up to 7 days following birth [3]. In contrast, the adult zebrafish heart undergoes a regenerative process following resection or cryo-injury which results in complete functional recovery [4]. The regenerative process in the adult zebrafish heart has been ascribed to dedifferentiation and proliferation of existing cardiomyocytes which demonstrate reactivation of a number of cardiac markers typically observed during early embryonic development [5].

While these models of cardiac regeneration are useful they have a number of limitations. The resection procedure is time-consuming, technically challenging and is associated with a modest mortality [6]. Both resection and cryo-injury models require up to 60 days for complete functional recovery. Therefore, a model of cardiac injury and repair that could be produced more easily using a high throughput approach with a shorter time for response and repair would complement these adult models.

The zebrafish embryo has a number of distinct advantages as an experimental model. Firstly, the heart adopts an adult configuration by 72 h post fertilisation (hpf) and can be clearly visualized using conventional microscopy by virtue of the embryo's high degree of transparency at this stage of development. Secondly, the embryo is readily manipulated by pharmacological and genetic techniques. Specifically, morpholino targeted-gene knock-down [7] allows rapid assessment of the roles of molecular pathways at a much reduced cost compared to similar approaches in the mouse.

Laser ablation techniques have been used in the zebrafish to induce kidney injury [8], thrombosis [9,10] and brain injury [11]. Laser injury of the atrio-ventricular cushion of the embryonic chick heart has been demonstrated but requires induction of bradycardia by thermal cooling to allow targeted injury [12]. The zebrafish embryonic heart is considerably smaller than the chick heart at equivalent developmental stages and is a more challenging structure in which to create precision injury [13].

Here, we present a technique which permits targeted and highly localized injury of the zebrafish embryonic heart at physiological heart rates. The precision is achieved by virtue of novel image-gating software which permits temporal synchronization between the cardiac cycle and delivery of the laser pulse. We demonstrate the functional and histological consequences of laser injury in different heart regions and we also provide evidence that the mechanism of recovery from laser injury to the ventricle is dependent on proliferation of cardiomyocytes.

## 2 Methods

### 2.1 Ethical approval

Authors certify that they comply with the Principles of Ethical Publishing in the International Journal of Cardiology. All experiments were approved by the local ethics committee and conducted in accordance with the United Kingdom Animals (Scientific Procedures) Act 1986 in an approved establishment.

### 2.2 Zebrafish maintenance

Zebrafish husbandry, embryo collection and maintenance were performed according to accepted standard operating procedures [14,15]. The cardiac myosin light chain 2:GFP transgenic line (tg(cmlc2:GFP)) [16] was used for all experiments, unless stated; embryos were maintained at 28.5 °C on a 14 h light/10 h dark cycle and staged according to Kimmel [17]. Embryos were kept in egg water until dechorionated and then in embryo medium [15]. All experimental procedures were performed at room temperature (RT, 23 °C).

### 2.3 Heart laser injury

**Fig. 1:**
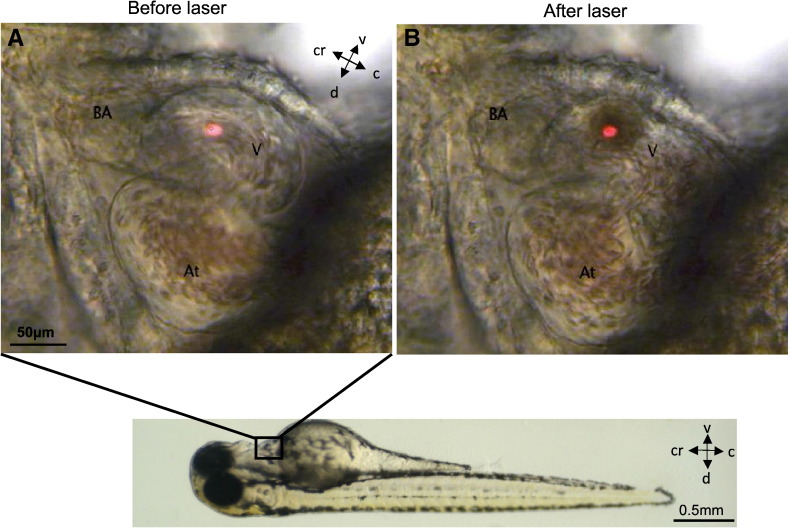
Zebrafish embryos 72 hpf (lower panel) were used for all experiments of laser injury. The laser pulse was delivered to the area of the ventricle indicated by the red dot (Panel A) and resulted in a clear burn-mark at the point of injury (Panel B), see also supplementary movie 1 (V – ventricle, BA – bulbus arteriosus, At – atrium). Position of the embryo is marked by compass lines (c-caudal, cr-cranial, d-dorsal, v-ventral).

A commercially available Infrared Laser Ablator XYClone (Hamilton-Thorne) normally used for injection of cells into the mouse blastocyst cavity was used. Embryos 72 hpf were anaesthetised using Tricaine 20 μM (Sigma-Aldrich) and were placed on their side on a plain glass microscope slide in a minimal amount of water (Fig. 1). Images were captured by a digital video camera mounted on a microscope and displayed on a monitor as a live video image of the sample. A built-in collimated red light beam target indicates the position of the laser beam in the field of view. The average energy delivered per pulse was 0.9 mJ over a duration of 3 ms (300 mW). The mid-cavity of the ventricle (supplemental video 1) was chosen as the target of injury as this region was readily and consistently injured in the majority of embryos without damaging surrounding cardiac and non-cardiac structures. After injury, each embryo was immediately placed in embryo medium to recover for a further 48 h. Controls embryos, kept in identical conditions and manipulated in the same way, received laser injury to the distal tail-fin.

### 2.4 Synchronization of the laser with the cardiac cycle

With the embryo lying on its side the atrium and ventricle are commonly overlapping making it difficult to injure the ventricle without damaging the atrium or other important cardiac structures. We addressed this problem using a novel approach by linking the laser to a personal computer (PC) containing custom-built software that permits temporal synchronization between delivery of the laser pulse and the phase of the cardiac cycle. The optical gating software is described in detail elsewhere [18] and was adapted in this case to provide a trigger signal for the laser to fire. The user selects an image in the cardiac cycle that the laser should be fired from a sequence of images. In our case, we chose the point at which the ventricle was at its largest diameter corresponding to mechanical end-diastole. The software then analyses subsequent image frames calculating the precise time at which the heart will next be in this position and the laser is automatically triggered to fire at this precise time point (supplemental movie 2). This approach also allowed us to assess the effects of delivering up to 5 laser pulses at 1 minute intervals to the mid-region of the ventricle without creating significant injury to the atrium, atrioventricular cushions or other cardiac structures. This avoided the occurrence of significant valvular regurgitation and prolonged conduction block which were observed in some cases using non-gated laser injury.

### 2.5 Assessment of heart function and tail blood flow

Video images of the beating heart were captured by video camera (IonOptix CCD100 MyoCam^tm^) mounted on a microscope Zeiss, Axioscope II MOT Plus) linked to a PC. Heart rate, ejection fraction and ventricle diastolic area were measured using image analysis software (ImageJ, NIH, Bethesda). Ejection fraction was estimated by subtracting ventricular systolic area from diastolic area, expressed as a percentage of diastolic area, as previously described [19].

Caudal Vein blood flow velocity was estimated in the posterior cardinal vein [20] by assessing frame by frame motion of single erythrocytes determined from video images. Four erythrocytes per fish (at least 4 embryos per group) over 10 frames at video frame-rate of 30 frames per second were analyzed using ImageJ to determine mean erythrocyte cell velocity (μm s^− 1^).

### 2.6 Exposure of embryos to aphidicolin

To assess the effect of cell cycle inhibition following laser injury, embryos were bathed from 60 hpf in embryo medium containing 150 μM aphidicolin (Sigma-Aldrich) solubilized in Dimethyl Sulfoxide (DMSO) 1% (Sigma). Laser injury was induced at 72 hpf and embryos maintained in aphidicolin for a further 48 h, until 120 hpf. Control embryos incubated in vehicle (DMSO 1%) from 60 hpf were treated in an identical manner with laser injury at 72 hpf. A control group of embryos exposed to aphidicolin with no laser injury was also included.

### 2.7 Zebrafish whole-mount immunostaining and histology

Embryos were euthanized in Tricaine 1 mM and fixed in 4% paraformaldehyde (PFA, Sigma) and hearts isolated by micro-dissection. These isolated hearts were pre-incubated in proteinase K (10 μg/ml), washed in PBS and Triton X100 (0.1%) and then Bovine Serum Albumin (5% for 3 h) before being incubated with anti Phospho-histone H3 antibody (Millipore 05-670; rabbit, 1:200), a marker of mitosis, followed by incubation with anti-rabbit antibody (Alexa fluor, Dako, 1:500). Subsequently, hearts were incubated in DAPI (Sigma, 1:1000), washed in PBS and then mounted in glycerol (100%). Confocal microscopy (Leica SP5) was used to capture z-stack images of isolated zebrafish heart ventricles at 3 μm intervals. The total number of ventricular cardiomyocytes (VC*t*) and the number of mitotic ventricular cardiomyocytes (VC*m*) were counted using ImageJ software, by marking each nucleus with a tag while moving progressively through the z-stack. Only cardiomyocytes were included in the counting process by ensuring that each nucleus was located within a GFP positive region of the heart. The atrium and bulbus arteriosus were excluded from counting. Counting was performed by a single individual (GM) and the intra-observer variation for a sample of 25 hearts was ± 4.5%.

Haematoxylin & Eosin (H&E) staining of whole embryos was performed at 2, 24 and 48 h post-laser injury and serial 4 μm sagittal sections were stained according to standard protocols [21].

### 2.8 Whole-mount TUNEL assay

Apoptotic cell death in whole-mount zebrafish was detected according to a modification of the ApopTag rhodamine In Situ Apoptosis Detection kit (Chemicon,Temecula, CA) protocol. Embryos were fixed in 4% paraformaldehyde (PFA) at 4 °C, washed in PBS, permeabilized with proteinase K (10 μg/ml) followed by two further washes in PBS. They were then fixed again in 4% PFA, then placed in prechilled ethanol:acetic acid (2:1) at - 20 °C washed in PBS-T (PBS 1X, 0.1% Tween-20) 3 times before incubation in equilibration buffer and further steps recommended by the manufacturer. TUNEL assay staining was quantified by counting positive staining puncta in the whole heart from z-stack confocal images using ImageJ.

### 2.9 Statistical analysis

Experiments were performed in triplicate with on average 20–30 embryos per experiment, unless otherwise stated. Data are presented as mean ± standard error of the mean (SEM). Statistical analyses were performed using GraphPad Prism 5. One-way or two-way repeated measures ANOVA followed by Bonferroni post-hoc test were used to compare means within and between groups. P values < 0.05 were considered significant.

## 3 Results

### 3.1 Effects of laser injury on cardiac performance

**Fig. 2:**
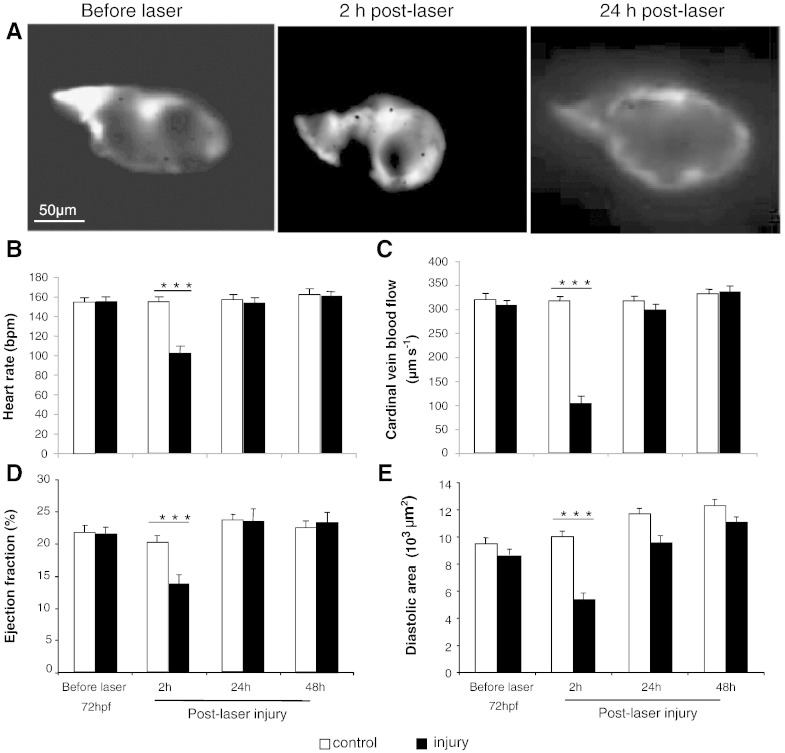
*Effects of a laser pulse to the ventricle on cardiovascular function.* Panel **A.** Images showing an in vivo zebrafish embryo heart ventricle before laser (72 hpf), at 2 and 24 h post-laser injury. Panels B-E Heart rate, cardinal vein blood velocity, ejection fraction and ventricle diastolic area assessed before laser and 2, 24, 48 h post-laser injury (Mean ± sem, N = 30 per group, 5 experiments, *** = p < 0.001, ANOVA test).

A single laser pulse to the mid-cavity of the ventricle (Fig. 1) resulted in instantaneous cardiac injury confirmed optically by a 2 to 4 s pause followed by marked bradycardia and a small amount of bleeding into the pericardial space. There then followed a gradual and progressive increase in heart rate (HR) over the following 2 to 3 min with a significantly reduced HR compared with controls by 2 h post-laser (117 ± 11 vs 157 ± 9 bpm, p ≤ 0.001, Fig. 2B). Laser injury also resulted in temporary cessation of caudal vein blood flow (CVBF) which partially recovered but remained significantly reduced at 2 h post-laser (103 ± 15 vs 316 ± 13 μm.s^− 1^, p ≤ 0.001, Fig. 2C). Ejection fraction (EF) was also significantly reduced at 2 h post laser (14.1 ± 1.8 vs 20.1 ± 1.3% p ≤ 0.001, Fig. 2A & D). The ventricle diastolic area (VDA) was also significantly reduced at 2 h post laser (5.3 ± 0.8 vs 10.1 ± 0.6 · 10^3^ μm^2^, p ≤ 0.001, Fig. 2A & E). These parameters recovered to control levels by 24 h and remained comparable to controls at 48 h post-laser. Increasing the number of laser pulses resulted in more severe damage to the ventricle with a pronounced fall in ejection fraction observed with 3 pulses at 2 h post-laser (Fig. 6). Ventricles treated with up to 2 laser pulses recovered fully while 3 laser pulses or more resulted in partial or failure of recovery of function by 48 h post-laser injury.

### 3.2 Effects of laser injury on cardiomyocyte proliferation

**Fig. 3:**
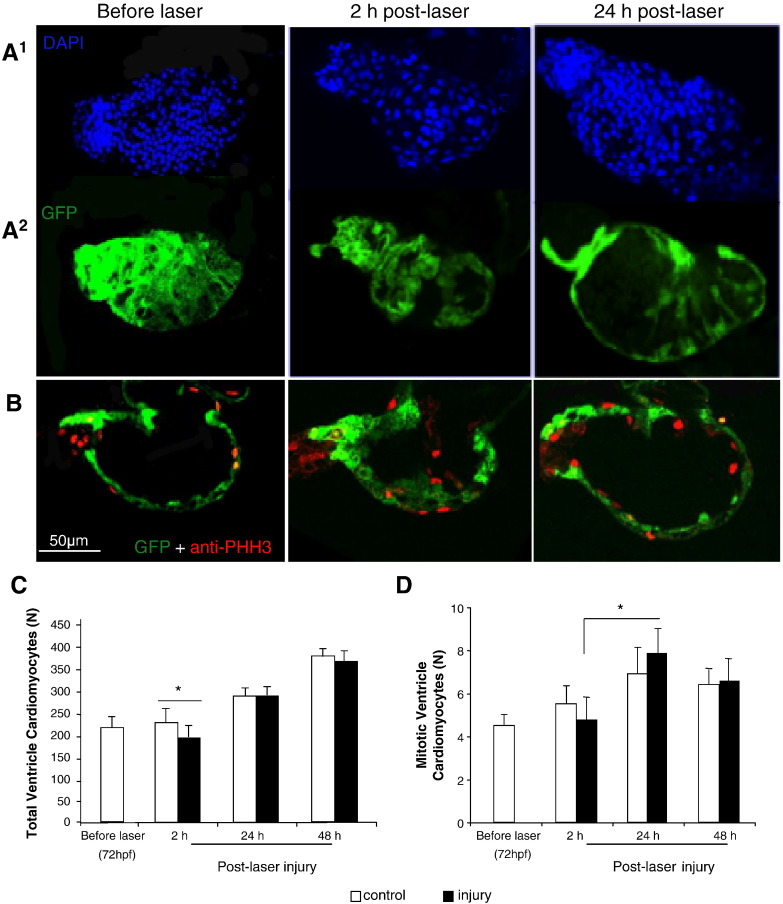
*Effect of a laser pulse on total and proliferating ventricle cardiomyocyte number.* Panel A^1^. DAPI stained nuclei counted as total number of ventricle cardiomyocytes (VC*t)* in cmlc2:GFP hearts, shown in the lower panel (A^2^). B. Phospho-histone H3 stained nuclei, in red, represent mitotic cardiomyocytes (VC*m)*. Total (C) and mitotic (D) ventricle cardiomyocyte number variations during normal development, from 72 to 120 hpf (48 h post-laser), and following laser injury (Mean ± sem, N = 28 per group, 5 experiments, * = p < 0.05, ANOVA test).

Control embryos demonstrated a progressive increase in total ventricular cardiomyocyte number (VC*t*) between 72 and 120 hpf consistent with normal development (Fig. 3A & C). At 2 h post-laser, we observed a statistically significant reduction in the number of cardiomyocytes in laser-injured ventricles (195 ± 15 vs 238 ± 15, p ≤ 0.05) which recovered to control levels by 24 h post-laser. Between 2 and 24 h post-laser we observed a mean 20% increase in cardiomyocytes in controls and a mean 49% increase in laser-injured ventricles. The number of mitotic cardiomyocytes (VC*m*), identified by anti Phospho-histone H3 (PHH3) staining at 2 and 24 h post laser was similar in controls (5.5 ± 0.6 vs 6.8 ± 1.0, p = 0.24) while it was significantly greater in laser-injured ventricles at 24 h post laser (4.7 ± 0.9 vs 7.9 ± 1, p = 0.04, Fig. 3B & D).

### 3.3 Effects of laser injury on ventricle apoptosis and structure

**Fig. 4:**
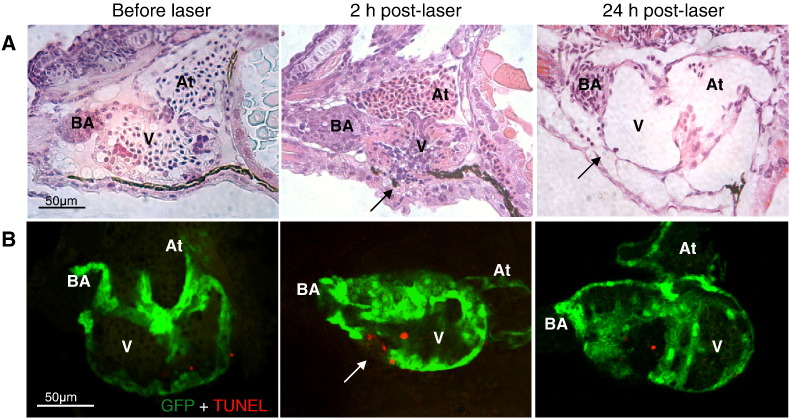
*Histological assessment of zebrafish heart ventricle laser injury.* Panel row A. Haematoxylin and Eosin staining showing apoptotic/necrotic bodies in the area of laser damage (black arrow) at 2 h post-laser injury and their absence at 24 h post-laser injury. Laser-injury also resulted in a reduction in thickness of the mural myocardium at 24 h post-laser (black arrow). Panel row B. Whole-mount zebrafish embryonic hearts stained with TUNEL assay, showing apoptotic bodies at 2 h post-laser (white arrows) in the area of laser injury. Few apoptotic bodies were detectable before laser or at 24 h post-laser in the site of injury (N = 6 per group). V – ventricle, BA – bulbus arteriosus, At – atrium).

In H&E stained sections there was evidence of focal fibrinoid necrosis of the pericardium and myocardium at 2 h post-laser as evidenced by the presence of amorphous hyper-acidophilic debris containing pyknotic and karyorrhectic nuclear remnants (Fig. 4A). Hearts treated with laser showed a greater number of apoptotic bodies, as shown by TUNEL assay (Fig. 4B) particularly around the region of the Laser burn at 2 h post-laser injury (Laser 5.2±1.0 vs controls 1.3±0.4). The number of apoptotic bodies had returned to control levels by 24 h post-laser. Mild haemorrhage and accumulation of eosinophilic proteinaceous oedema fluid was present within the pericardial space (Fig. 4A). At 24 h post injury although active necrosis was absent there was moderate segmental reduction in thickness of the mural myocardium compared to controls with multifocal adhesions of the pericardium to the thoracic wall. In fish receiving 3 or more laser pulses there were residual foci of necrosis and more extensive cardiac / thoracic wall adhesions; these features were not observed at 48 h post-laser injury.

### 3.4 Cell cycle inhibition with aphidicolin

**Fig. 5:**
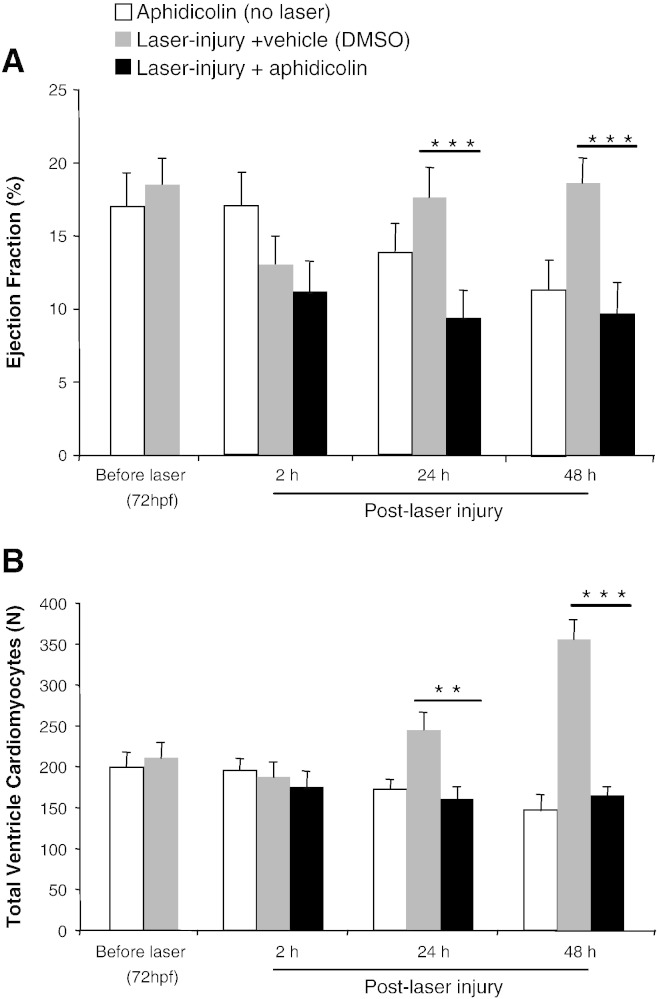
*Effects of cell cycle inhibition on cardiac recovery following laser injury.* Aphidicolin (150 μM) inhibited reduced ejection fraction (A) and ventricle cardiomyocyte number (B) in zebrafish embryos in non-lasered hearts and following laser injury (mean ± sem, N = 18 per group, 3 experiments, * = p < 0.05, ** = p < 0.01, ANOVA test) while lasered hearts incubated in DMSO (1%) showed full recovery by 24 h following laser.

**Fig. 6:**
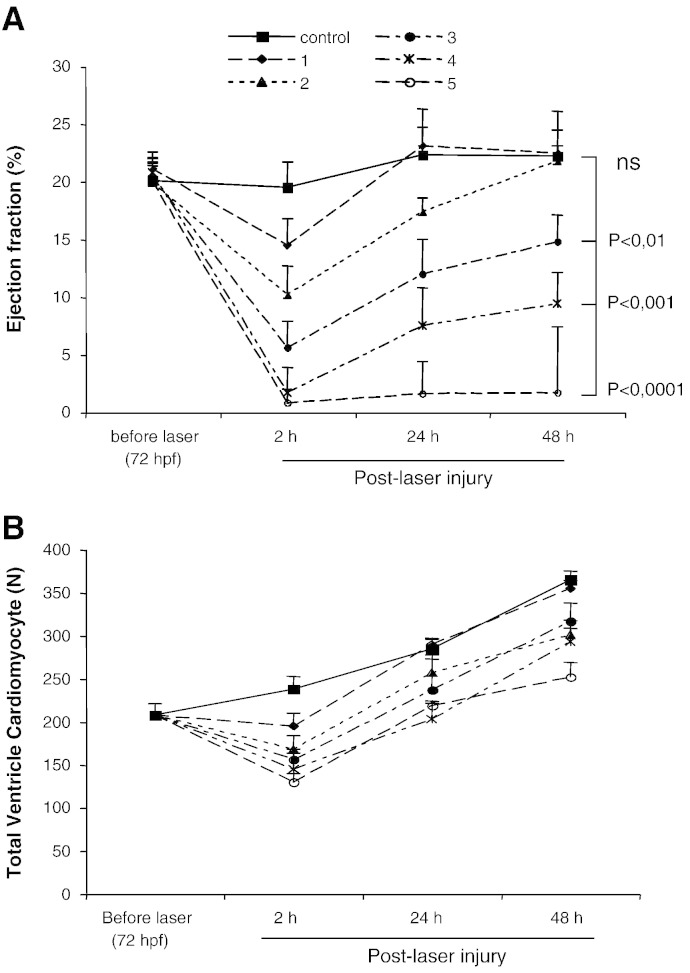
*Effects of multiple laser pulses on the zebrafish embryo heart ventricle.* Effects of multiple laser pulses on ejection fraction (A) and total ventricle cardiomyocyte number (B) at 2, 24 and 48 h post-laser injury. Ejection fraction at 48 h post-laser remained significantly reduced following 3 or more pulses compared with controls (mean ± sem, N = 16, 3 experiments, ANOVA) while it had fully recovered in hearts treated with 1 and 2 laser pulses.

Incubation of embryos in the cell cycle inhibitor aphidicolin (Fig. 5) caused a progressive reduction in ejection fraction over 48 h (Fig. 5A). In hearts exposed to aphidicolin and injured with laser, ejection fraction fell further and failed to recover while laser-injured hearts exposed to vehicle recovered ejection fraction to control values by 24 h post-laser (9.1 ± 2.1% vs 18.8 ± 2.3%, p ≤ 0.001). This lack of functional recovery in aphidicolin treated hearts was accompanied by a reduced number of ventricular cardiomyocytes (VC*t*) consistent with a failure of proliferation (Laser + Aphidicolin vs Laser + vehicle, 169 ± 19 vs 342 ± 18 at 48 h, p ≤ 0.001, Fig. 5B).

## 4 Discussion

Laser ablation of the zebrafish embryonic heart represents a novel model of highly localized injury to the ventricle. The strengths of this model are the simplicity by which injury is produced, the rate at which individual animals can be processed and the ease with which pharmacological and genetic interventions could be tested to assess their impact on the response to, and recovery from, laser injury. Furthermore, this is a model of in-situ cellular injury in a vertebrate animal with many neuroendocrine and molecular pathways similar to those found in higher vertebrates. While it is clearly different from mouse or rat models of injury it has distinct and significant advantages over experimental models that involve isolated cardiomyocytes [22], cell culture [23] or other isolated physiological cardiac preparations such as Langendorff-perfused hearts.

To our knowledge, this is the first report of laser injury to the heart in the zebrafish embryo. We have also shown that the physiological consequences of laser injury can be quantified using simple microscopy combined with standard image analysis techniques.

In order to produce highly precise injury to the ventricle without damaging surrounding structures we had to overcome the problem of the anatomical relationship between the atrium and ventricle which are typically overlapping in the zebrafish embryonic heart with the fish lying on its side. Our initial approach was to reposition the embryo to ensure there was minimal overlap of cardiac structures and thus provide a clear target for the laser. This was time consuming and commonly resulted in stress or injury to the embryo. We therefore adapted a previously validated video-image synchronization technique [18] and by linking this to the laser we were able to produce precisely targeted injury to very specific regions of the heart at selected points in the cardiac cycle. The synchronisation system was simple to use, highly accurate and allowed a high throughput of up to 50–60 embryos per hour. The only comparable high-throughput model reported in the literature in the zebrafish embryo is a transgenic chemical cell ablation model in the zebrafish embryonic heart [24] which results in diffuse cardiomyocyte death. The laser injury model presented here results in regional damage of a nature similar to that resulting from ligation of a coronary artery in mammals.

### 4.1 Zebrafish embryonic ventricle recovers following laser injury

Significant injury was immediate as demonstrated by cardiac arrest and cessation of tail blood flow (supplemental movies 1 and 2). This was followed by a significant fall in heart function followed by complete recovery by 24 h. We observed a loss of ventricle cardiomyocytes consistent with cell death and supported by histological changes suggestive of necrosis. Apoptosis also contributed to cardiomyocyte loss following injury as demonstrated by TUNEL assay revealing increased numbers of apoptotic cells at 2 h post laser almost exclusively present in the region of cardiac injury. Notably, increased apoptosis is associated with early phases of regeneration as shown in several organisms [25,26]. Programmed cell death selectively localized in the area of laser injury may also play a role in triggering regenerative responses observed in the zebrafish embryonic heart in our model.

### 4.2 Cardiomyocyte proliferation drives the functional recovery of Laser-injured ventricles

Accurate counting of all cardiomyocytes in the developing ventricle was assessed by counting DAPI positive nuclei within the GFP positive region of the ventricle. This technique has been used previously [27] and is acknowledged to be an accurate method to assess proliferation of cardiomyocytes. In our model, cardiomyocyte number increased by 20% in control ventricles between 72 and 96 hpf as part of normal development. During this same time-period, we observed a greater increase in the number of cardiomyocytes in laser-injured ventricles by almost 50% suggesting that the proliferative process had been accelerated by laser injury. Enhanced proliferation of cardiomyocytes was further supported by significantly more PHH3 nuclei in laser-injured ventricles. The underlying mechanism for this apparent acceleration of cardiomyocyte proliferation is possibly related to laser-induced release of local factors enhancing proliferative change in surviving cardiomyocytes or could be due to circulating factors released in response to abnormal peripheral circulation. A further intriguing observation is that ventricles treated with multiple laser pulses also showed significantly greater increases in cardiomyocytes compared to controls at 24 and 48 h post-laser despite the lack of recovery in ejection fraction. Indeed, our observations are consistent with findings in the adult zebrafish [4] and the mouse [28] where cardiac injury has been shown to trigger epicardial cell proliferation. Further evidence to indicate that proliferation of new cardiomyocytes contributed to functional recovery of the laser-injured ventricle was provided by the experiments showing that the cell cycle inhibitor aphidicolin effectively inhibited cardiomyocyte proliferation and functional recovery following cardiac laser ablation. These findings suggest that the repair and recovery mechanism following laser injury is dependent on processes driving the formation of new cardiomyocytes [29] although we cannot completely exclude cardiomyocyte hypertrophy and an epicardial cell role as contributing at least in part to the recovery process.

## 5 Conclusions

This study has demonstrated the feasibility and reproducibility of a novel and highly precise method of injury to the embryonic zebrafish heart using a laser. With this approach, we have shown, for the first time, the ability of the zebrafish embryo heart to recover from injury by a mechanism largely dependent on the proliferation of new cardiomyocytes. Furthermore, laser injury appeared to stimulate this proliferative process. This model could be used to study novel cellular and molecular mechanisms associated with cardiac injury and repair using a high throughput approach.

The following are the supplementary data related to this article.

Movie 1 *Laser pulse injury (without synchronisation) of the zebrafish embryonic heart ventricle at 72 h post-fertilization***–** A single laser pulse, using the XYClone Laser Ablator, to the ventricle of a zebrafish embryo (72 hpf) results in instantaneous cardiac injury associated with marked bradycardia and gradual recovery of cardiac rhythm over the next few minutes. A laser burn-mark is clearly seen in the wall of the ventricle. This is an example where there is a clear view of non-overlapped cardiac chambers.

Movie 2 *Laser pulse injury using the synchronization software of the zebrafish embryonic heart ventricle at 72 h post-fertilization*. In this example, atrium and ventricle are overlapped. Attempting to injure the ventricle with a non-synchronized laser system would result in damage to adjacent structures. Synchronizing the laser pulse with the cardiac cycle allows highly precise and targeted injury to the ventricle at end-diastole and consequently minimizes damage to surrounding structures.
