# Supplementary figures and images for: Laser-targeted ablation of the zebrafish embryonic ventricle: A novel model of cardiac injury and repair
Source: Int J Cardiol. 2013 Oct 9;168(4):3913–9. doi: 10.1016/j.ijcard.2013.06.063 (PMC3819623; doi:10.1016/j.ijcard.2013.06.063)

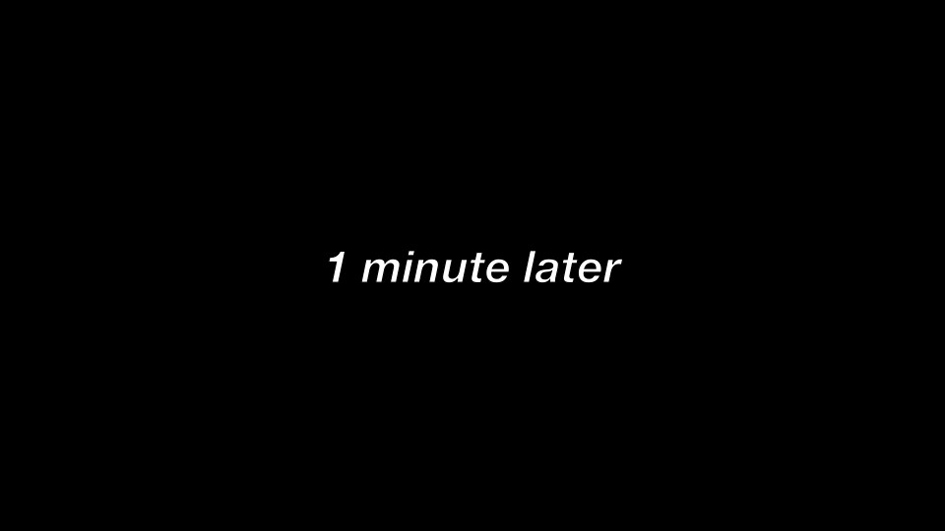

Supplement: Movie 1 — Laser pulse injury (without synchronisation) of the zebrafish embryonic heart ventricle at 72 h post-fertilization– A single laser pulse, using the XYClone Laser Ablator, to the ventricle of a zebrafish embryo (72 hpf) results in instantaneous cardiac injury associated with marked bradycardia and gradual recovery of cardiac rhythm over the next few minutes. A laser burn-mark is clearly seen in the wall of the ventricle. This is an example where there is a clear view of non-overlapped cardiac chambers. [file mmc1.jpg]

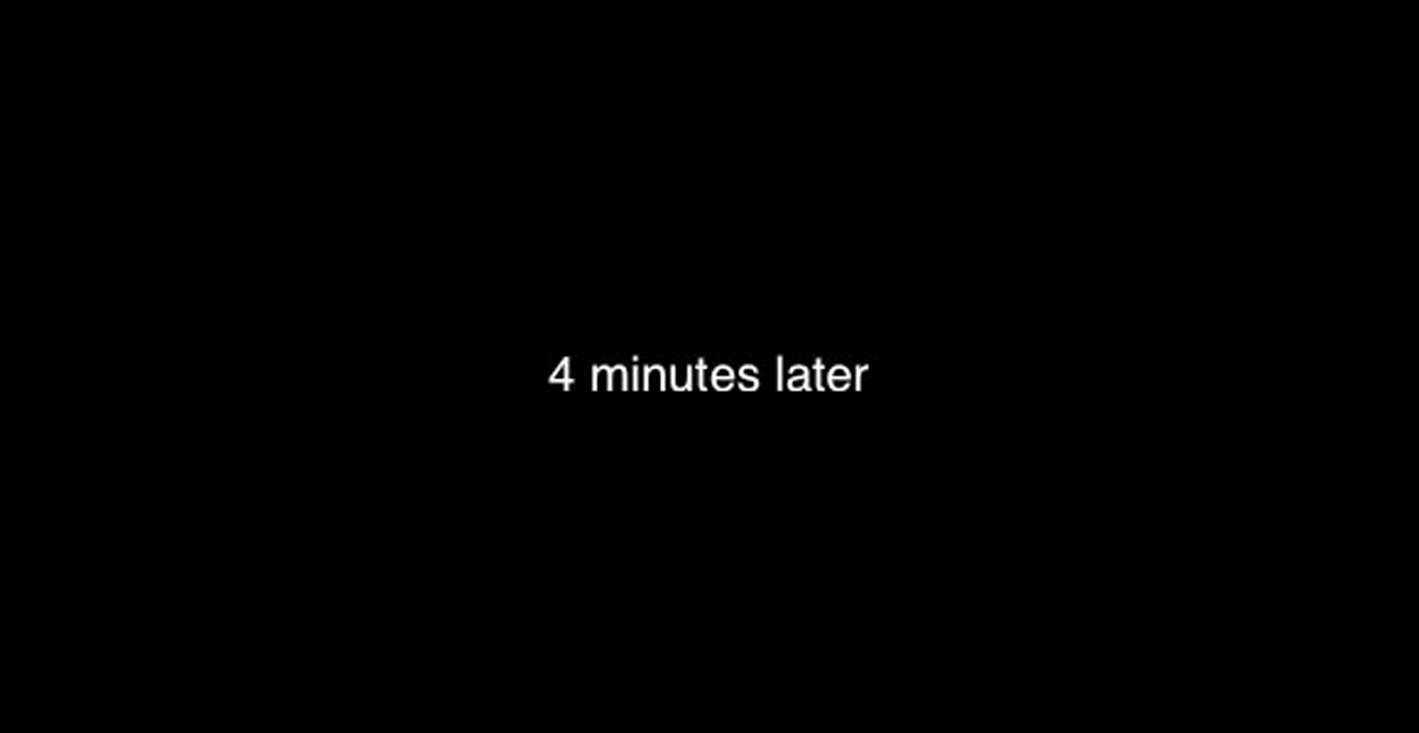

Supplement: Movie 2 — Laser pulse injury using the synchronization software of the zebrafish embryonic heart ventricle at 72 h post-fertilization. In this example, atrium and ventricle are overlapped. Attempting to injure the ventricle with a non-synchronized laser system would result in damage to adjacent structures. Synchronizing the laser pulse with the cardiac cycle allows highly precise and targeted injury to the ventricle at end-diastole and consequently minimizes damage to surrounding structures. [file mmc2.jpg]
